# Complicated Complication: How Interventional Radiologists Should Manage Acute Iatrogenic Cardiac Tamponade

**DOI:** 10.7759/cureus.3708

**Published:** 2018-12-08

**Authors:** Taylor S Harmon, Gregory Wynn, Travis E Meyer, Daniel Siragusa, Jerry Matteo

**Affiliations:** 1 Radiology, University of Texas Medical Branch, Galveston, USA; 2 Radiology, University of Florida College of Medicine, Jacksonville, USA

**Keywords:** interventional radiology, pericardial effusion, cardiac tamponade, computed tomography, pericardiocentesis, percutaneous pericardial catheter, decompensation, iatrogenic injury, complicated complication, interventional cardiology

## Abstract

Computed tomography guided biopsies are common interventional procedures that are necessary for confirmation of imaging findings. Additionally, percutaneous biopsies are necessary for the elucidation of a patient’s clinical findings with disease pathology. Though interventionists perform these procedures regularly without consequence, various complications may arise depending on the tissues biopsied. Examples of such complications may include hemorrhage, hematoma formation, or perforation of surrounding vessels. In the case of mediastinal biopsies, less common but higher-risk complications may include pneumothorax, perforation of arterial vessels, and damage to the cardiac tissues resulting in decompensation. Interventionists should understand the risks of performing these procedures and should be prepared to intervene if life-threatening complications arise. As interventional cardiologists are often prepared to manage decompensating cardiac complications, interventional radiologists must likewise anticipate the same to occur when conducting procedures that may affect cardiac tissues. The following case demonstrates a technique for correcting a complicated complication arising from a mediastinal biopsy, which resulted in a pericardial effusion and patient decompensation.

## Introduction

Acute pericardial effusions are ominous in nature and result from traumatic, pathologic, or iatrogenic causes. There are degrees in severity of acute pericardial effusions that range from asymptomatic and clinically insignificant to effusions that result in cardiac tamponade and patient decompensation [1]. Usually, these complications are iatrogenic and inadvertently caused in cardiac catheterization laboratories while cardiac interventions are being conducted. One documented retrospective report showed that out of 150 patients with cardiac tamponade relieved by pericardiocentesis, the most common etiology was iatrogenic [2]. For interventional cardiologists it is important to recognize the clinical signs and symptoms of pericardial effusions that lead to patient decompensation. Furthermore, management of these insidious complications is equally paramount in stabilizing the patient.

Clear guidelines for triaging and managing pericardial effusions within the cardiac catheterization laboratory have been well documented [3-5]. It is common protocol in most cardiac catheterization laboratories to have pericardial puncture “tray set-ups” for when procedural complications arise [1]. Though it is traditionally a part of cardiac catheterization laboratory protocol to prepare for acute cardiac complications and patient resuscitation, the interventional radiology procedure suites lack these measures. For acute pericardial effusions that result in decompensation, interventional radiologists must be prepared in the same manner that cardiologists are when the same complications arise. The following case demonstrates a computed tomography (CT) guided mediastinal biopsy complicated by an acute pericardial effusion and patient decompensation. Using conventional interventional methods, the interventional radiologist performed life-saving measures to correct the inadvertent cardiac collapse.

## Case presentation

**Figure 1:**
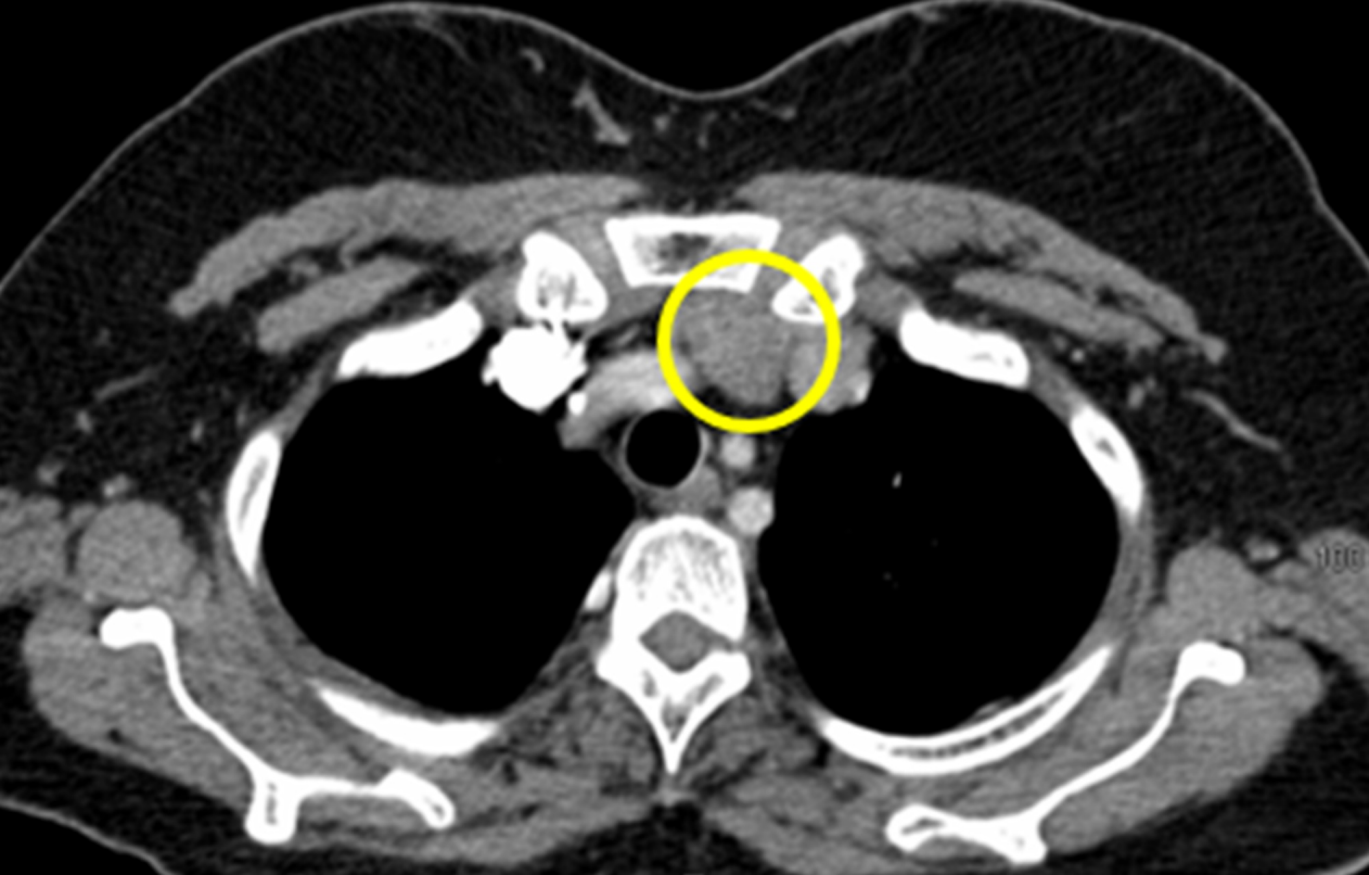
Axial Computed Tomography of the Chest Revealing an Anterior Mediastinal Mass The figure shows an axial computed tomography of the chest, revealing an incidental anterior mediastinal mass (yellow circle).

A 47-year-old-female with shortness of breath had an incidental anterior mediastinal mass that was discovered on chest CT (Figure 1).

**Figure 2:**
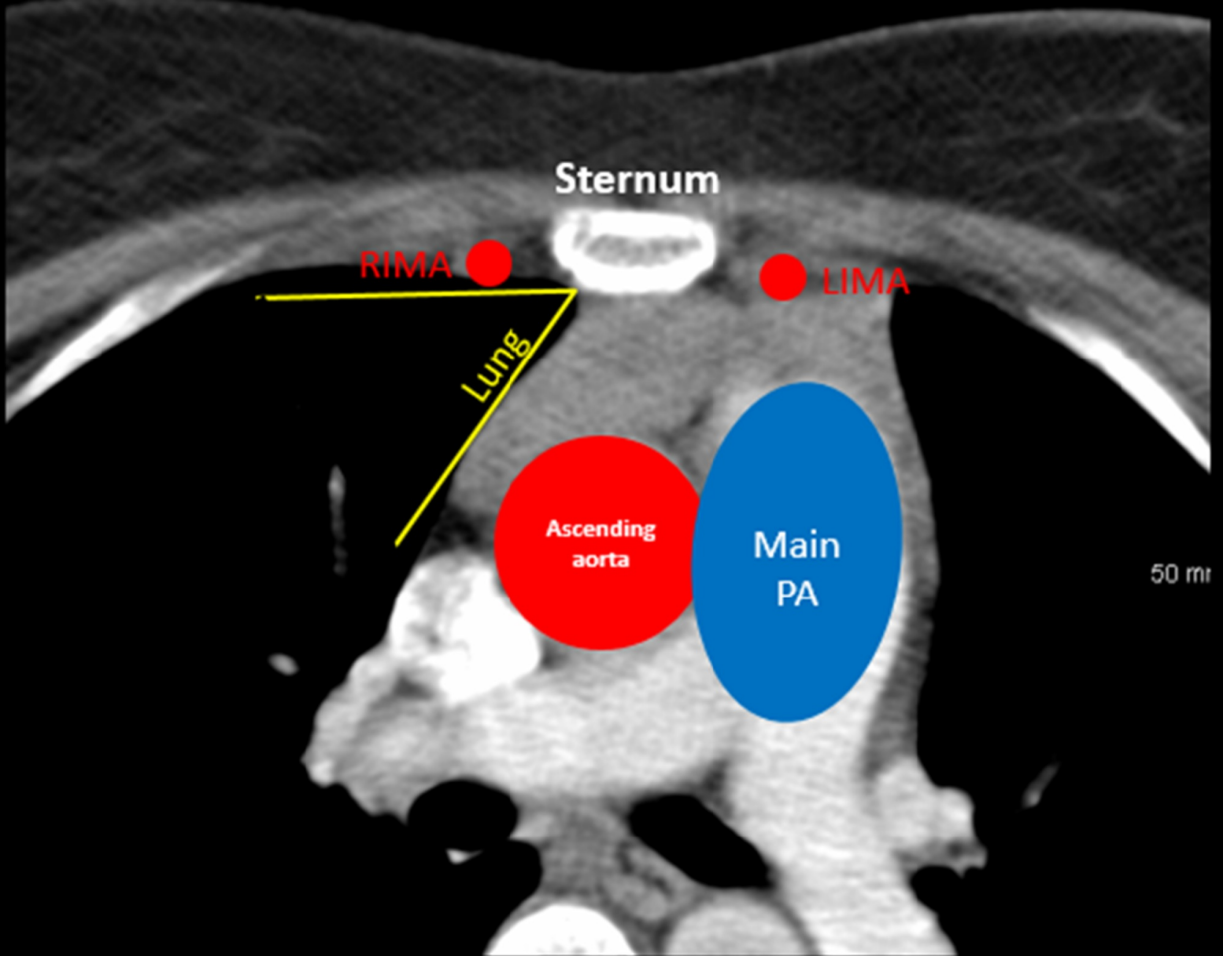
Anatomical Depiction of Vital Structures Surrounding an Anterior Mediastinal Mass The computed tomography of the chest shows the difficulty the interventional operator would have when attempting to biopsy the anterior mediastinal mass. The sternum is the most anterior structure to the mediastinal mass. Lateral to the sternum on both sides are the left (LIMA) and right (RIMA) internal mammary arteries (smaller red dots), and they are both anterior to the mediastinal mass. The lungs are shown bilaterally, represented by the black areas, with the border of the right lung shown. Both lungs are lateral to the mediastinal mass. The main pulmonary artery (PA) and ascending aorta are posterior to the anterior mediastinal mass.

It was decided that the mass should be biopsied for identification of cell type. However, several surrounding structures, including the sternum anteriorly, left internal mammary artery laterally, main pulmonary artery and aorta posteriorly, and right lung laterally, made it difficult to access the mass (Figure 2).

**Figure 3:**
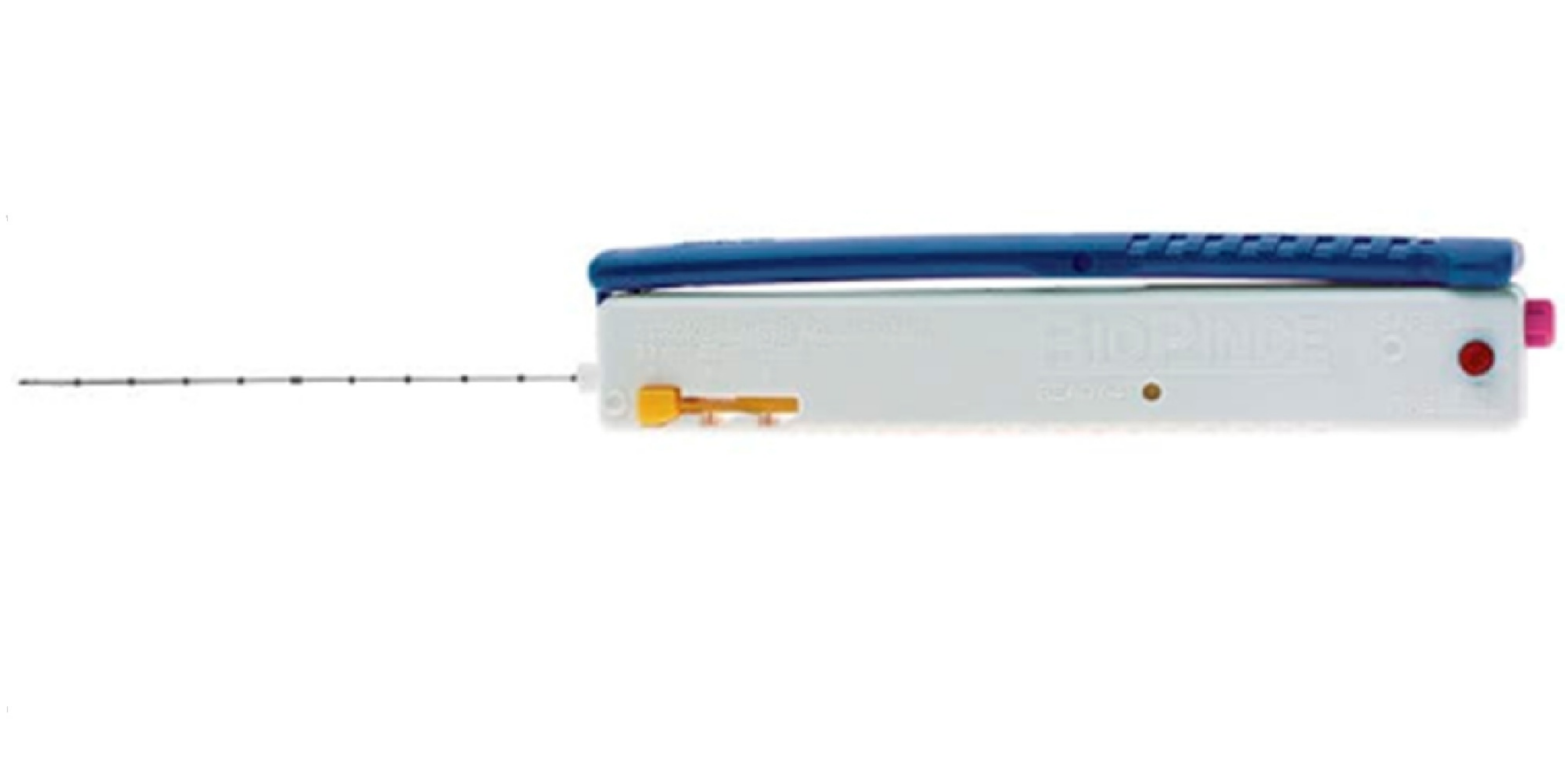
BioPince® Biopsy Device A BioPince® biopsy device was used to take samples of the anterior mediastinal mass during the procedure.

A BioPince® (Argon Medical Devices, Frisco, Texas) biopsy device was used to sample the mass (Figure 3).

**Figure 4:**
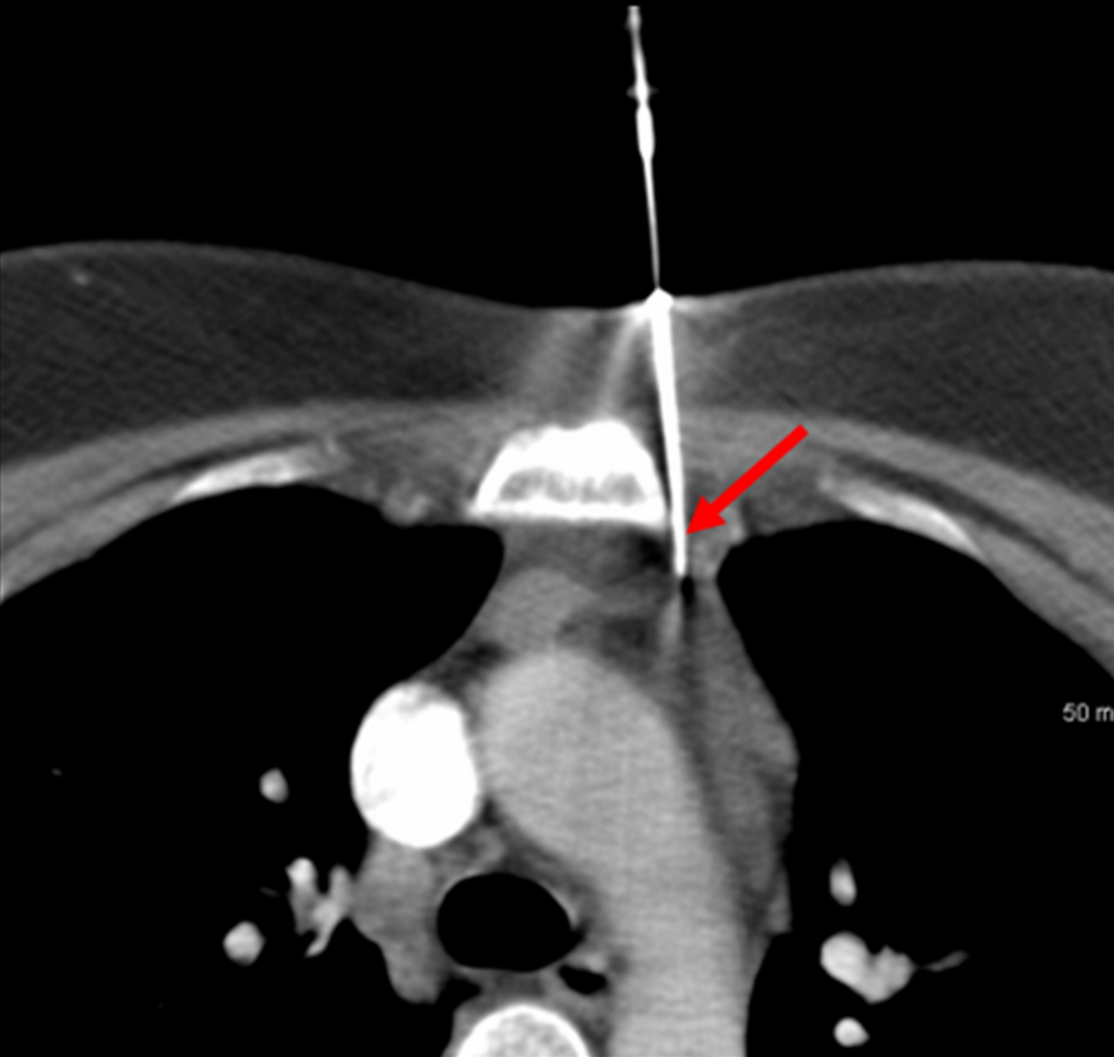
Biopsy of the Anterior Mediastinal Mass Using a Parasternal Approach The biopsy needle device was inserted just lateral of the left sternum using a parasternal approach (red arrow). This was determined to be the safest and most effect means for biopsying the anterior mediastinal mass.

A parasternal approach was used to access the mass just lateral to the sternum on the left (Figure 4).

**Figure 5:**
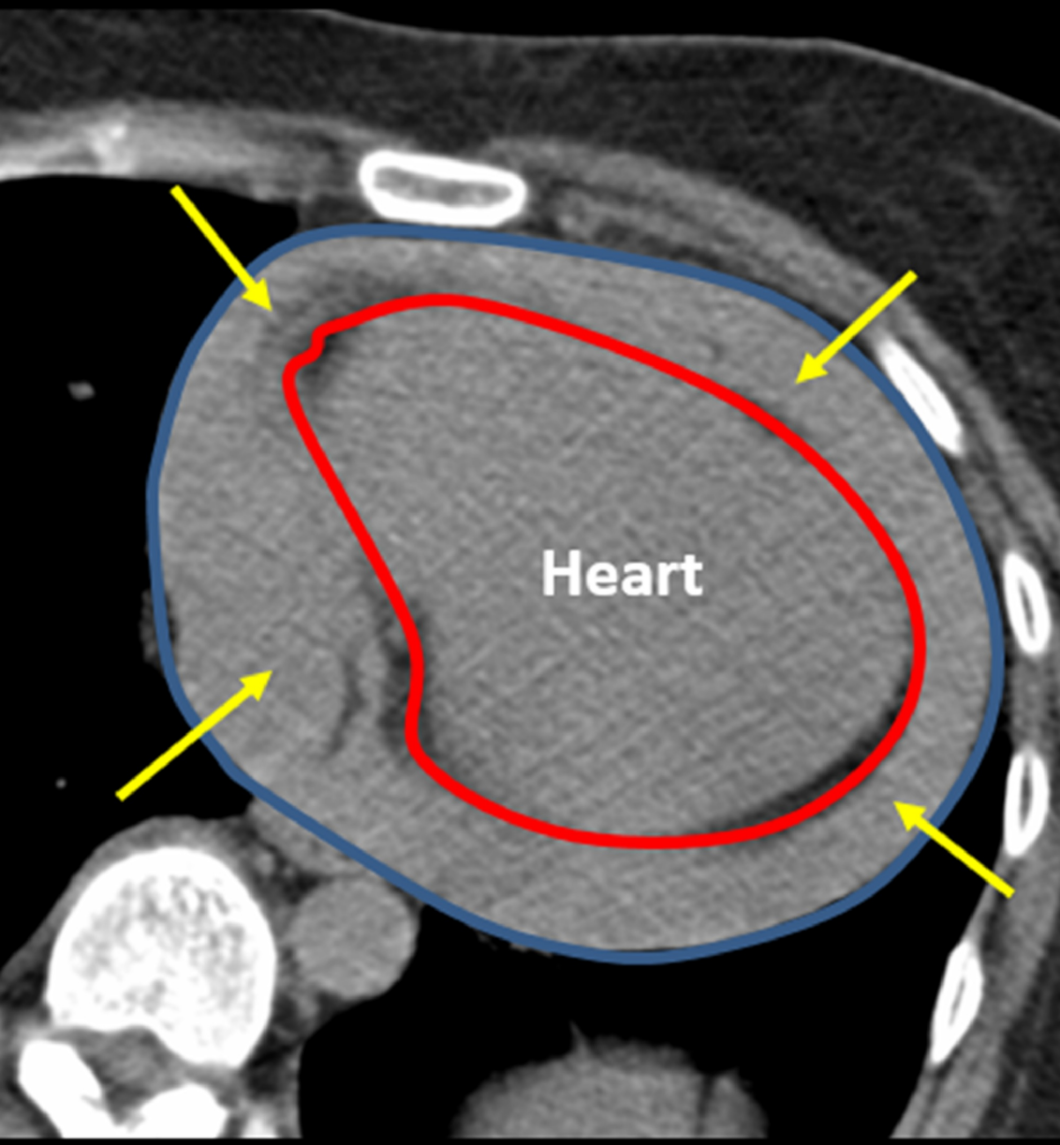
Post-biopsy Acute Cardiac Tamponade A computed tomography of the axial chest after taking several samples of the anterior mediastinal mass shows acute cardiac tamponade. The blood collection can be visualized within the pericardial space (yellow arrows), between the heart (red outline) and the pericardium (blue outline).

Three 18 gauge 2.3 cm core biopsy specimens were passed off to cytopathology for immediate analysis. The patient then reported to have acute shortness of breath, chest tightness, diaphoresis, along with decreased blood pressure associated with cardiac tamponade. A repeat CT image of the chest revealed copious amounts of blood within the pericardium (Figure 5).

**Figure 6:**
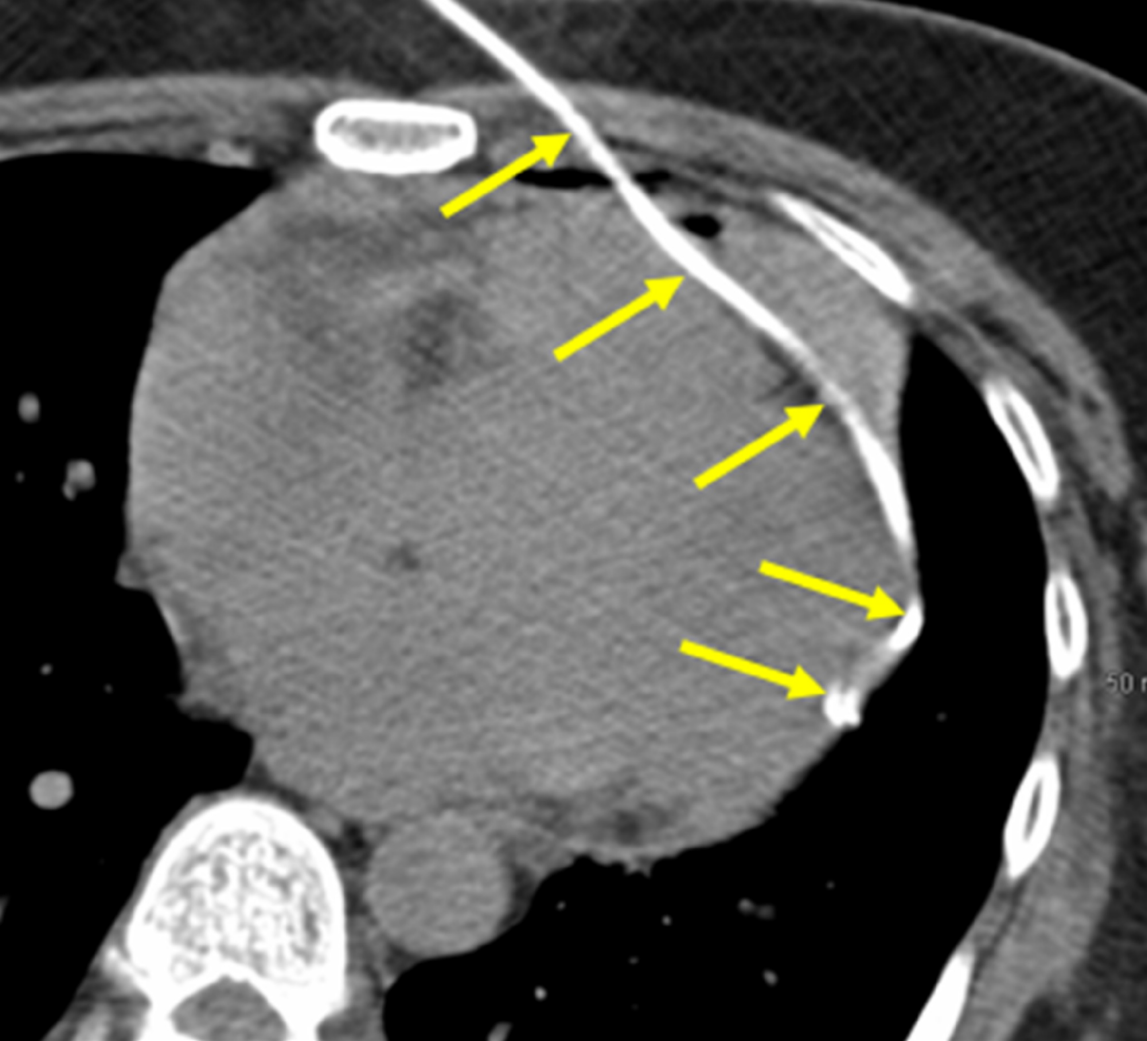
Percutaneous Pericardial Catheter Placement The interventional operator placed a 6 French pig tail catheter within the pericardial space to relieve the gathering blood (yellow arrows).

The interventional operator quickly decided to place a six French pig tail catheter within the pericardium to drain the blood in the pericardium (Figure 6) and 180 ml of unclotted pericardial blood was rapidly removed.

**Figure 7:**
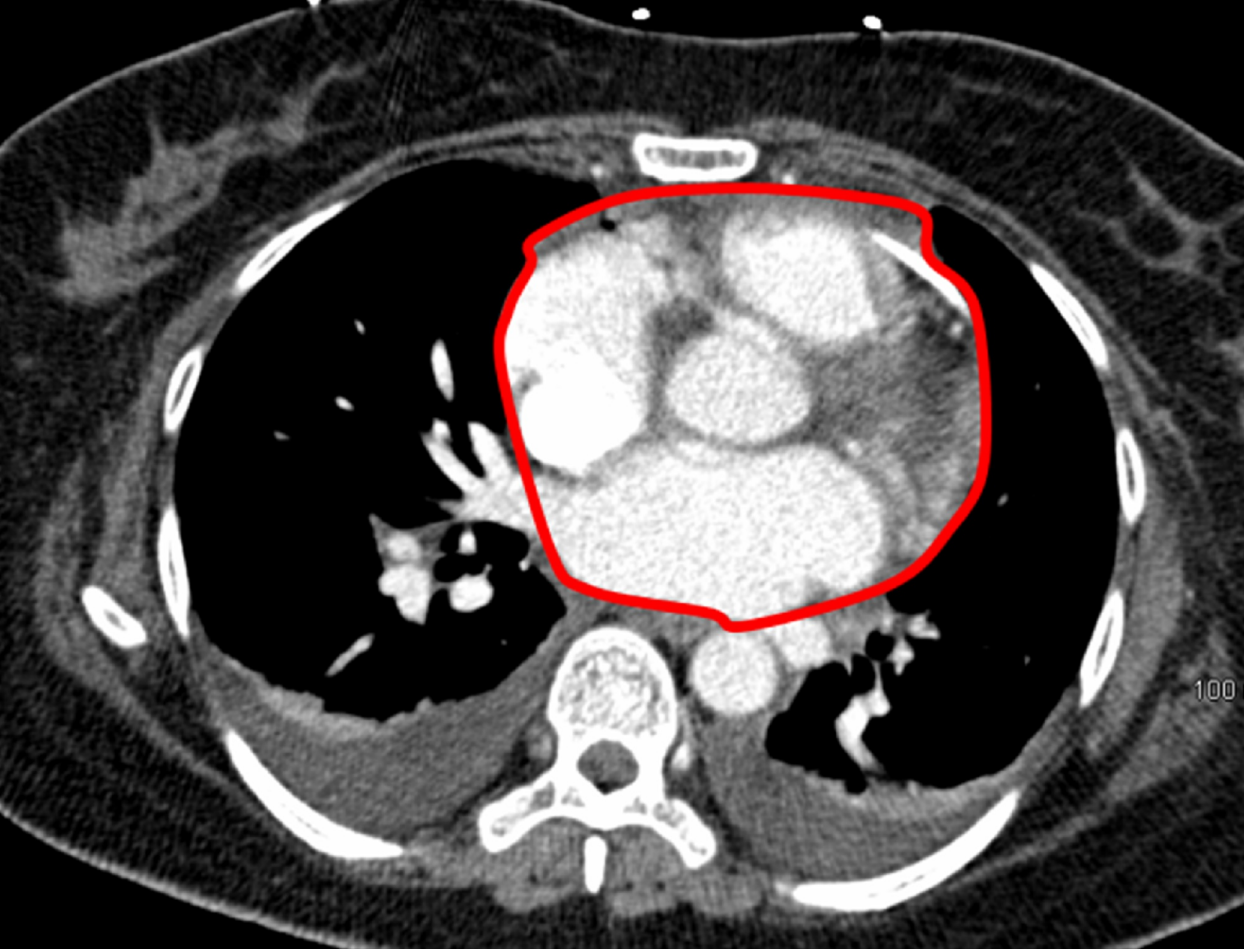
Post-procedural Follow-up Computed Tomography Angiogram of the Chest A 48-hour post-procedural follow-up axial computed tomography angiogram of the chest shows resolution of cardiac tamponade. The computed tomography angiogram shows the lack of blood within the pericardial space, with a homeostatic heart border (red outline).

The patient’s vitals were stabilized and the patient further reported relief of her symptoms. The pigtail catheter was connected to a low suction drainage bulb, which demonstrated slow continuous drainage. Drainage ceased after 24 hours with a total output of 300 mls. Forty-eight hours later, a CT angiogram showed no significant pericardial effusion remaining (Figure 7).

The pigtail catheter was removed and the patient was discharged home at 72 hours.

## Discussion

Severe cardiac complications are uncommonly managed within the spectrum of interventional radiology. However, this does not negate the importance of managing such life-threatening events when they arise. When an interventional radiologist must manage a complicated complication as in the preceding case, they must act upon their interventional training to resuscitate their patient. Conveniently and as frequently managed by interventional cardiologists, radiologists can adapt their interventional training to improvise and treat cardiac complications equally in capability.

Other than pericardiocentesis, percutaneous pericardial catheter drainage with catheters, such as the pig tail catheter, have been reported to successfully evacuate pericardial fluid many times before [6-7]. Though considered conventional interventional procedures in cardiology, both pericardiocentesis and percutaneous pericardial catheter drainage for pericardial effusions are not intuitively encompassed by interventional radiology. However, there have been reports of pericardiocentesis and percutaneous drainage of pericardial fluid performed by interventional radiologists in controlled, non-emergent settings [8].

Only one case of pericardiocentesis conducted by an interventional radiologist as an insidious emergency has been documented [9]. In this specific emergent case, the interventional radiologist was performing catheter guided thrombolysis of a saddle embolus when the operator had to perform a pericardiocentesis, subsequent to iatrogenic pericardial effusion. As in the preceding case presented, the interventional radiologist made unanticipated decisions utilizing their interventional skillset as competent and similar to a cardiologist in a similar situation. The result was the stabilization of the patient and a complete recovery.

Well-documented triage reports also suggest that interventional management may not be the standard of care when managing patients with acute pericardial effusions [3-5]. Specifically, there are patients that require surgical management by cardiothoracic surgery who fail interventional management and continue to decompensate [3-5]. Interventional radiologists should not only be aware of the iatrogenic complications that can arise, but take calculated measures in settling these unforeseeable errors. Furthermore, this may require consulting the proper surgical team after interventional management of these complicated complications have failed. In the preceding case, the operator was able to place a percutaneous drain within the pericardium to drain the fluid and reconstitute the patient. However, surgical assistance should have been consulted if the intervention did not resolve the pericardial effusion or patient decompensation. 

The preceding case calls attention to procedural protocol as the sudden precipitous decline of a patient, due to an unexpected cardiac complication, should be anticipated by interventional radiologists. Especially in the setting of procedures that involve the possibility of damaging cardiac tissues, as is the case of mediastinal CT-guided biopsies, interventional radiologists should be prepared to intervene upon their patients who are exposed to iatrogenic injury. As interventional cardiologists have separate pericardiocentesis trays at their disposal, it would be beneficial for interventional radiologists to have similar set-up entities if needed [1]. Interventional radiology technologists should also be informed and prepared to assist the operator if such complications arise.

## Conclusions

Interventional radiologists perform a diversity of procedures throughout all organ systems, which are all specific for complications that vary in severity. Of the procedures they perform, some of the most insidious and complicated to correct are those that effect cardiac tissues. As presented in the preceding case, mediastinal CT-guided biopsies can expose the cardiac tissues to iatrogenic injury, resulting in acute pericardial effusion and patient decompensation. As interventional cardiologists are trained to prepare for intervention upon these complicated complications in the cardiac catheterization laboratory, interventional radiologists should do the same, especially to avoid poor patient outcomes. Interventional radiologists should amend the procedural protocols in the event of such cases, so that the interventional management team is prepared and patient outcomes are preserved.
